# Mycotoxin Detection Plays “Cops and Robbers”: Cyclodextrin Chemosensors as Specialized Police?

**DOI:** 10.3390/ijms9122474

**Published:** 2008-12-05

**Authors:** Pietro Cozzini, Gianluigi Ingletto, Ratna Singh, Chiara Dall’Asta

**Affiliations:** 1 Molecular Modeling Laboratory, Department of General and Inorganic Chemistry, University of Parma, Via G.P. Usberti 17/A 43100, Parma, Italy. E-Mails: gianluigi.ingletto@unipr.it (G. I.); ratna.singh@nemo.unipr.it (R. S.); 2 INBB, National Institute for Biostructures and Biosystems, Viale Medaglie d’oro 305, 00136 Rome, Italy; 3 Department of Organic and Industrial Chemistry, University of Parma, Via G.P. Usberti 17/A 43100, Parma, Italy. E-Mail: chiara.dallasta@unipr.it

**Keywords:** Chemosensing, fluorescence, docking, molecular engineering

## Abstract

As in a cops and robbers play we discover new mycotoxins and metabolites everyday and we are forced to develop new molecules quickly as chemo- or biosensors or to modify existing molecules able to recognize these new hazardous compounds. This will result in an enormous cost saving to agro-food industry through the prevention and reduction of product recalls and reduced treatment costs. Here we present a brief review of the rapid methods used to detect mycotoxins, considering usefulness and limits. Then we propose a new fast, efficient and cheap methodology, based on a combination of computer chemistry aided design and fluorescence, that can help to drive synthesis in a more efficient way.

## 1. Introduction

Since improvement of “life quality” is one of the most important objectives of global research efforts, in the last decade the production–processing–distribution chain has to be carefully checked “from field to fork”, quality of life being closely linked to food quality and safety.

Among the most prominent hazards for consumer health, food pollutants such as xenobiotics (e.g. mycotoxins, xenoestrogens, phytoalexins) and residues (e.g. pesticides, dioxins, polychlorinated aromatic hydrocarbons, phenils) are of serious concern for the food industry (http://www.efsa.europa.eu/EFSA/efsa_locale-1178620753812_KeyTopics.htm).

In particular, in the last years there has been increasing awareness of the hazards imposed on both human and animal health by mycotoxins present in food and feed. In the last years the European Union has revised the EU legislation about mycotoxins in order to harmonize the EU Members’ national laws, to enforce new limits and to regulate a wider variety of commodities. The EU legislator emended regulations not only for food but also for feed in order to improve farm animal health [1].

The chemical diversity of mycotoxins and the wide range of matrices in which they can be found pose great challenges to analytical chemists. Therefore, a need exists for a reliable, economical and easy-to-use assay for the measurement of the mycotoxin content, especially in the raw materials for food and feed production. Moreover, the fact that mycotoxins are often found as traces leads to the need for very sensitive techniques, able to detect the toxins at ppb, or even ppt, levels [2].

Therefore, there is a real need for rapid, sensitive and inexpensive sensors for the detection of toxic compounds along the food processing chain to obtain real-time monitoring data on contamination, which may be use for food safety assessment. This will result in an enormous cost saving to agro-food industry through the prevention and reduction of product rejections.

Recognition-based techniques are potential candidates to fulfill many of the above requirements, due to their general high selectivity and sensitivity. Among these methods, great efforts have been made for the development of immunochemical methods for mycotoxin detection [3, 4]. Although traditional immunoassay-based methods such as Enzyme-linked immunosorbent assays (ELISA), which allow parallel analysis of multiple samples, have been widely applied to mycotoxin detection, ELISA methods are still time-consuming and suffer from false positive and/or false negative results. With the aim of simplifying procedures and developing robust, portable analytical tools, the recent trend is to focus on immunodipsticks and immunosensors, which overcome the lengthy analysis time required by ELISA assays.

## 2. Bio- and chemosensing in food diagnostic: fundamentals

Recognition methodologies are based on the use of a sensor, which is a receptor able to selectively bind a target compound and to affect its chemico-physical properties as a consequence of binding. The change resulting from the interaction between the target (guest) and the receptor (host) can be converted into a quantifiable signal from a transducer, which is mainly electrochemical (potentiometric, amperometric, conductimetric), optical (absorbance, fluorescence, chemiluminescence), thermal or piezoelectric devices. The receptor can be a bioactive macromolecule, such as an enzyme or an antibody (biosensors), or a supramolecular host, such as cyclodextrins, crown ethers or imprinted polymers (chemosensors) the former commonly known as biosensors and the latter as chemosensors [5]. The screening devices usually applied in food diagnostic are actually immunosensing methods as they mainly involve enzyme- or antibody-based approaches [6]. So far, only few studies have reported the use of supramolecular devices for chemical recognition in food and environmental analyses.

## 3. Fluorescence: some base principles

An adequately excited system (atoms, molecules, ions) undergoes a transition from the GES (Ground Electronic State) to an EES (Excited Electronic State). In many aromatic organic compounds, when excited by UV radiation, the transition is from the lowest vibrational level (the more populated at RT) of the GES to excited vibrational levels of the first EES. When the excitation ceases, the organic molecule releases the excess energy and returns to the GES spontaneously. This can occur in different ways. The radiative emission of radiation from an EES to the GES is called luminescence, which is divided in fluorescence and phosphorescence. Fluorescence is the most common emission in solution and phosphorescence is observable especially in the solid phase [7].

In fluorescence, the transition is “spin allowed” and occurs between singlet states. The GES and EES are “singlet states” as all the electrons have opposite spins (ΔS(total spin)=0 in both the states, ΔS=0) and the total electron spin selection rule ΔS=0 is followed. The lifetime of the EES is very short (10^−4^ - 10^−9^ sec) because the transition is allowed. In phosphorescence, the transition is “spin forbidden”, and occurs between the triplet EES and the singlet GES. The GES, as described above, is a “singlet state”, but the EES is a triplet state as 2 electrons in two different M.O. have parallel spins (ΔS=1, ΔS=1) and the rule ΔS=0 is broken (in the following, we will explain how this prohibition can be partially overcome). The lifetime of the EES is long (from 10^−3^ sec to several minutes) since the transition is forbidden.

The triplet EES, whose energy is lower than the singlet EES, cannot be reached directly from the GES, but only from the singlet EES. In fact, one of the processes that competes with fluorescence in aromatic organic molecules, is “intersystem crossing” or “change of multiplicity” in the excited state: 2 antiparallel electronic spins turn into 2 parallel spins. In this way a transition from a singlet EES to a triplet EES occurs (ΔS=1) and the selection rule ΔS=0 is broken. This is possible because of a perturbation (S.O. coupling) due to an interaction between two magnetic fields connected to the orbital motion of the electron (orbital angular momentum) and the electron spin (spin angular momentum) and the resulting mixing of the two angular momenta. Therefore the S.O. coupling (enhanced in the presence of heavy atoms like P and S) is the cause both of the intersystem crossing and phosphorescence, because in both the transitions the ΔS=0 rule is violated.

What happens when the excitation ends? The excited molecule collides with the surrounding molecules and, for this reason loses energy non-radiatively and decays into the lowest vibrational level of the EES (t ∼ 10^−12^ sec). In solution, the solvent molecules re-orientate around the excited molecule (t ∼ 10^−11^ sec). Now, if the surroundings cannot absorb the large energy excess necessary to decay from the EES to the GES, the molecule drops from the lowest vibrational level of the EES to an excited vibrational level of the GES and the excess energy is released as a photon in the range of VIS or UV (t ∼ 10^−15^ sec). Following the radiative transition, the molecule releases to the lowest vibrational level of the GSE (t ∼ 10^−12^ sec) and, in solution, the solvent molecules rearrange themselves around the deactivated molecule (t ∼ 10^−11^ sec). The reason for the shift to longer wavelengths of emission with respect to absorption (Stokes’ shift) is clear, taking into account how the excitation and the deactivation occur.

The mechanism of fluorescence suggests that, in solution, the emission intensity depends on the capacity of the solvent molecules to absorb vibrational quanta. When the solvent is made up of molecules with widely spaced vibrational levels (e.g. H_2_O), they can accept the large quantum of electronic energy and therefore quench the possible radiative emission. Fluorescence quenching refers to any process that causes a decrease in the emission intensity and here we will consider two of the most common types of quenching: “collisional quenching” and “static quenching”.

Both collisional and static quenching involve a molecular contact between a fluorescent molecule (fluorophore) and molecules that cause quenching (quencher). In collisional quenching, collisions occur between a fluorophore and a quencher, that makes up its environment (e.g. H_2_O) and the fluorophore then decays nonradiatively to the GES. In static quenching, a non fluorescent complex between fluorophore and quencher is formed.

In addition to luminescence and to non-radiative processes that compete with it (e.g. quenching of various type) a non radiative energy transfer (Fluorescence Resonance Energy Transfet or FRET) is possible from an electronically excited molecule D* (donor) to a non excited molecule A (acceptor) when:

A is close to D* and a coulombic interaction (strictly dependent on the distance between D* and A) exsists
the energy of an EES of A is lower than the EES of D* and there is an overlap between the D* emission spectrum and the A absorption spectrum.

Therefore, FRET, that is particularly important due to its applications, is a radiationless mechanism of energy decay as no photon is transferred from D* to A. The entire process can be briefly represented as follows: D* (in an EES) A (in the GES) → D (in the GES) A* (in an EES). FRET is experimentally confirmed when fluorescence intensity and lifetime of D* decrease and fluorescence of A (if it is fluorescent) increases. The D* intensity fluorescence decreases in the presence of A, because a part of its energy is transferred to A, instead of beeing emitted as a photon. The lifetime of D* decreases as the energy transfer to A is another pathway for the EES to decay to the GES.

The interest in emission spectroscopy, in particular fluorescence, is essentially due to its sensibility, simplicity and to the wide range of information that can be obtained. It is therefore possible, and of major interest, to reveal fluorescent systems, even in the case of low intensity and very low concentrations, for example aflatoxins in the environment or in food products.

## 4. Fluorescence as technique of choice for contaminant detection

The selectivity of the sensing system for the target analyte is mainly determined by the recognition element (host), which is able to interact selectively with the target (guest). On the contrary, the sensitivity of the sensor is greatly influenced by the transducer. For this reason, the most successful diagnostic techniques are based on optical immuno- and chemosensing: different optical transduction methods have been used to develop immunosensors, such as surface plasmon resonance (SPR) [8], total internal reflection ellipsometry (TIRE) [9], optical waveguide light spectroscopy (OWLS) [10]. However, despite recent advances in these optical detection techniques, fluorescence is undoubtedly the most powerful. In most of the fluorescence-based methods recently reported in the literature, the biorecognition molecule is not in intimate contact with the transduction element and the fluorescence is measured in solution, thus they cannot be considered proper biosensors. For this reason, immobilization of the antibody on an optic fiber is necessary [11, 12]. Since many mycotoxins such as aflatoxin, zearalenone and ochratoxin A, show a natural fluorescence, fluorescence-based techniques can be directly applied. Nonetheless, a labelling step with a proper fluorophore can be implemented prior to the use of fluorescence techniques for non-fluorescent mycotoxins such as trichothecenes or fumonisins.

## 5. Rapid detection of mycotoxins in food

### 5.1. Immunosensors

Immunosensors are devices based on the detection of specifical analyte–antibody interactions which are responsible for changes in physicochemical parameters. These changes can be easily sensed using suitable electronical devices [4, 13]. Immunochemical methods for mycotoxin detection have been recently extensively reviewed [14–17].

The first immunochemical approach for mycotoxin analysis was the development of immunoaffinity based columns (IAC), commonly used for sample cleanup [18]. The immunoaffinity column is packed with monoclonal antibodies coated on the surface of spherical beads. When a diluted sample extract is applied to the column, the analyte is specifically bound by the antibody, while most of the potentially interfering material is washed out. The toxin is then eluted using a denaturating solvent such as methanol or acetonitrile. Despite their high selectivity, a main drawback is that these columns cannot be easily reused, due to a fouled column, denaturation/renaturation of antibody, and leaching of antibody strongly affect the reliability of the system. Recently, microbead assays have been developed by miniaturizing the IAC assays. These devices allow for a one-step analysis, since cleanup and detection can usually be performed on the same instrument [19].

#### 5.1.1. Colorimetric and luminescent sensors

Colorimetric and luminescent sensors are based on a characteristic spectral behaviour of the analyte, since its UV-VIS absorption or emission under specific conditions is transformed into the analytical signal. Among these techniques, fluorescent sensors for mycotoxin determination are more frequently reported. In particular, the natural fluorescence of the analyte can be used for a direct non-competitive assay, whereas a direct competitive assay requires the use of a proper fluorescent label. Finally, the indirect competitive format is based on labelled fluorescent antibodies. In comparison with the colorimetric sensor, fluorescent labelling speeds up the assay time by eliminating the need for substrate addition and colour development [20]. Traditionally, fluorescent sensors allowed detection of one single target, but recently a biosensor array has been developed for simultaneous, multiplexed detection of OTA, FB1, AfB1 and DON [21–23].

The development of multianalyte assays seems to be possible, allowing the determination of both large and small molecular weight food contaminants simultaneously, as recently reported by Sapsford *et al*. [24]. Among the immunoassay formats, non-competitive methods are the simplest and fastest although they are suitable only for fluorescent compounds. These methods are based on the binding of target mycotoxins without competition with a labelled molecule. Since the target fluorescence is directly detected, the response of the sensor is directly proportional to the analyte concentration. Until now this approach has been used for AFB1 detection [11], but many mycotoxins have their own fluorescence (i.e. aflatoxins, OTA, ZEA, citrinin), thus non-competitive assay could be potentially applied to multianalyte analyses.

#### 5.1.2. Surface Plasmon Resonance Sensors

Surface plasmon resonance (SPR) is the basis of many standard tools for measuring adsorption of material onto planar metal (typically gold and silver) surfaces or onto the surface of metal nanoparticles. Surface plasmons are surface electromagnetic waves that propagate in a direction parallel to the metal/dielectric (or metal/vacuum) interface. Since the wave is on the boundary of the metal and the external medium (air or water for example), these oscillations are very sensitive to any change of the boundary, such as the adsorption of molecules to the metal surface. This principle allows the direct detection of the biological interaction without the labelling any of the interactive systems. In their simplest form, SPR reflectivity measurements can be used to detect analytes observing the changes in the local refractive index upon adsorption of the target molecule to the metal surface [25]. Immunoassay formats for the SPR sensor are similar to those used for indirect competitive ELISA assay. Since most mycotoxins have a low molecular mass, the mass change caused by the binding to the surface may be too small to result in a significant change in refractive characteristics. Therefore, indirect methods with antigen-modified surfaces have been mostly used for mycotoxin quantification [26, 27].

In the case of SPR sensors, as for other sensor types, one of the critical characteristics is the regeneration process [8]. If the antibody has high affinity, the regeneration solution cannot completely remove the bound antibody from the sensor surface immobilized with hapten–protein conjugates. Application of single-chain antibody fragments instead of antibodies seems to be an interesting strategy to simplify the regeneration procedure and make regeneration conditions less stringent [28]. Contrary to luminescent sensors and common immunoassay methods, the SPR approach does not require special labels and therefore non-specific interactions may occur and interfere under conditions of real sample analysis. Thus, additional clean-up steps are required. In any case, since no signal enhancement is included in SPR, test sensitivity is relatively low compared to enzyme or fluorescence immunoassays.

#### 5.1.3. Fluorescence polarization immunoassay

Fluorescence Polarization Immunoassay (FPIA) is a type of homogeneous competitive fluorescence immunoassay. With competitive binding, antigen from the specimen and antigen-fluorescein (AgF) labeled reagent compete for binding sites on the antibody. As a homogeneous immunoassay, the reaction is carried out in a single reaction solution.

Larger molecules rotate more slowly in solution than smaller molecules. This principle can be used to distinguish between the smaller antigen-fluorescein molecule (AgF), which rotates rapidly, and the larger antybody bound-antigen-fluorescein complexes(Ab-AgF), which rotate slowly in solution.

When polarized light is absorbed by the smaller AgF molecule, the AgF has the ability to rotate its position in solution rapidly before the light is emitted as fluorescence. The emitted light will be released in a plane different from the absorption plane. In the case of the larger sized Ab-AgF complex, the same absorbed polarized light is released as polarized fluorescence because Ab-AgF complex does not rotate as rapidly in solution. The light is released in the same plane of space as the absorbed light energy, and the detector can measure it.

Polarization measurements may be performed using portable equipment. Sometimes interaction kinetics can be relatively slow and the incubation time can greatly affect the assay sensitivity. FPIAs have been developed for deoxynivalenol [29–31], fumonisin [32], aflatoxins [33], ochratoxin A [34] and zearalenone [35].

### 5.2. Non instrumental methods

In the last decades a great number of rapid methods for mycotoxin detection have been reported. In particular, non-instrumental rapid screening techniques show an increasing interest, since they could be used outside the laboratory environment providing immediate results. Quantification of the toxin level, indeed, is not always required: a simple presence/absence test can be sufficient for raw commodities, avoiding delay in further processing steps.

Since the non-instrumental estimation of results rely on visual evaluation, colorimetrical or luminescent interactions are commonly used, such as enzymes for catalytic enzymatic reactions, colloidal gold, fluorescent labels and liposomes, encapsulating a visible dye. These kits provide a yes/no response due to the presence of the target analyte in concentrations higher than the fixed cutoff level, allowing for rapid on-site screening of food and giving results easily interpretable by nonspecialists. Some tests indicate semiquantitative estimation based on the colour intensity, but usually semi-quantification requires a reader for colorimetric measurements.

#### 5.2.1. Lateral flow devices

Immunochromatographic assays, also called “Lateral Flow Tests” are user-friendly formats requiring a very short time for results. Strips thus provide reliable testing that might not otherwise be available in third world countries.

Basically, a ligand that can be bound to a visually detectable solid support, such as dyed microspheres, can be qualitatively tested and in many cases even semi-quantitatively. The two predominant approaches to the tests are the direct non-competitive and the competitive reaction schemes. After application on the conjugate pad, the liquid components of the assay move along the membrane by capillary flow to the absorbent pad. Additional chemicals or handling steps are not required. The most popular label for the lateral flow test is colloidal gold consisting of particles with a 40-nm diameter which provide red-coloured binding zones. The test line is coated on the membrane with analyte-carrier protein conjugate. To simplify interpretation of the result, a control line is used as a secondary zone, coated with secondary antibody, above the test line. Liquid sample material or sample extract, applied on the sample pad, dissolves colloidal gold-labelled specific antibodies and carries them along the membrane.

When target analyte is present in the sample, the specific antibodies bind it. The whole complex migrates along the membrane and binds to the secondary antibodies on the control line. If the analyte is absent, the labelled specific antibodies bind to the analyte–protein conjugate on the test line. Therefore, absence of analyte results in red colour both for test line and control line. In the presence of target analyte above the cutoff value only the control line is red.

Lateral flow devices with colloidal gold labels have been studied for the most important mycotoxins, such as aflatoxins, DON, T-2 toxin, fumonisin, OTA and ZEA [36, 37]. Assay sensitivity can be improved by using a portable hand-held instrument for quantification. A major hindrance to the development of reliable lateral flow devices for mycotoxins is the toxins’ low molecular weight. Unlike the detection of larger antigens, where multiple antibodies can be used to make noncompetitive formats, the detection of low molecular weight toxins still relies upon competitive assays.

#### 5.2.2. Dipstick tests

Dipstick or strip or dot-ELISA assays are usually based on a membrane with a spot of the specific antibody .Several assay steps are required. According to the direct competitive ELISA scheme, the dipstick with attached antibody is placed in different solutions containing the sample, the analyte–enzyme conjugate and then the chromogenic substrate. Several dipsticks have been described in the literature for trichotechenes and fumonisin [38–40]. The application of dipsticks to multi-analyte analysis of mycotoxins has also been reported, although the sensitivity of the multiple assay seemed to be lower than for single analytes [41].

#### 5.2.3. Flow-through devices

The flow-through assay for mycotoxins is based on a direct competitive ELISA format, where specific antibodies are attached to a membrane placed on an absorbent body. The sample is added to the upper surface of the membrane where it flows through the membrane into the pad of absorbent material while analyte binds to the antibody spot on the membrane. Then the analyte– enzyme conjugate is added and bound by the remaining unbound antibodies. The last step is the chromogenic substrate application to obtain the coloured product in the presence of enzyme. Flow-Through devices have also been reported for several mycotoxins, such as ochratoxin A [42–44], aflatoxin M1 [45], T2 toxin [46] and fumonisin [47]. As reported by Schneider *et al*. [40], dipstick and flow-through formats for FB1 showed the same sensitivity in buffer solutions but were 50 times less sensitive than the corresponding microtitre plate ELISA with instrumental detection. To increase the sensitivity of flow-through devices some modifications should be applied, such as the modification of the membrane and the development of a different spotting method [48]. Multi-toxin analysis was successfully proposed for Flow-through devices and applied to the determination of seven mycotoxins: AfB1, FB1, T-2 toxin, roridin A, DON, diacetoxyscirpenol and OTA [49].

## 6. Chemosensensing systems

Only a few chemosensors for food analyses have been described in the literature, although chemical recognition has advantages over biological recognition systems, such as low cost, robustness and stability for long storage periods. The main drawback of chemosensing is the lower selectivity for the guest when compared to antibody-based approaches.

As recently reported by several authors [50, 51], strong efforts have been made in the last years in replacing natural biomolecules with artificial biomimic receptors. Different approaches have been developed, such as combinatorial synthesis of molecular receptors, combinatorial library of nucleic acids (aptamers) and molecular imprinted polymers (MIP). These receptors can be designed to have the required specificity for a chosen analyte or a whole class of target analytes.

Since biomimetic tecniques allow to overcome the problem of low stability of antibody, observed in extreme environments such as pH, organic solvents and high temperature, the development of synthetic receptors based on mimic techniques has also been recently introduced into the field of mycotoxin detection. In particular, many studies have recently been carried out for the development of MIP-based methods for food diagnostic [52, 53].

MIPs are synthetic receptors with high-affinity sites that can selectively recognize a target analyte, based on its shape, size or functional group distribution. Receptors prepared by using molecular imprinting are promising due to their easy preparation, thermal and chemical stability, and long shelf-life at room temperature and humidity [54]. Thus MIPs are considered an excellent alternative for clean up and pre-concentration of samples containing contaminants such as mycotoxins [55]. The use of molecularly imprinted polymers as solid-phase extraction for the selective concentration and clean up of different compounds has been reported in the literature [56–60].

The limitations of the approach are due to the lack of tracers closely resembling the analytes and the high cost and toxicity of mycotoxins, which cannot be used as imprited template and should be replaced by a synthetic mimic. These drawbacks compromise the affinity and, as a consequence, the sensitivity of the device. Future research is required, as currently available MIPs are not selective enough to compete with natural biorecognition receptors.

A different approach, based on chemosensing, involves the use of “smart molecules” showing biomimetic properties towards mycotoxins. These compounds could exhibit a substrate-selective recognition mechanism similar to that of antibodies or enzymes, while being more stable and affordable. The development of such biomimetic compounds requires a strong synthetic effort based on a sound molecular design to allow a target-tailored approach.

Molecular modelling, a powerful tool to implement conformational study of molecules such as drugs, proteins, macromolecules etc., has been applied to the design of synthetic receptors since it allows computational chemists to generate and refine molecular geometry, driving synthesis in feed-back.

Molecular modelling is particularly important in application for two aims: the comprehension of experimental data in absence of structural information and the design and the optimization of new compounds to drive new synthesis.

In this paper we propose a fast, efficient and cheap methodology based on a combination of computer chemistry aided design and fast fluorescence, that can help to drive the synthesis of smart chemosensors in a more efficient way.

## 7. The proposed paradigm

Computer aided techniques in chemistry (also called Computer Aided Chemistry), are well developed to simulate physico-chemical characteristics of a new molecule, to study the energetics of a complex, or to explain the behaviour of a molecule in lack of structural information, in order to define a reaction model from experimental data.

In case of food pollutants the availability of new cheap chemosensors is of paramount importance. To reach this goal it is necessary to focus on selective, inexpensive, fast and productive synthesis that could lead to produce the exact molecule simulated with modeling.

Lipkowitz [61] reviewed many computational studies of guest inclusion in cyclodextrins (CDs) over the past several decades. Molecular dynamics and molecular mechanics calculations, based on Newtonian force fields, are the most common methods applied with the exception of QSAR models. In recent times AM1 methods has been applied to the complex AFB1-β-CD trying to explain the fluorescence process [62].

Our protocol is based on a docking/scoring technique using a docking package, GOLD and a scoring functions, HINT, as a post processor. Using an internal scoring functions of GOLD plus HINT, we obtain a consensus scoring approach. HINT, a “natural” force field, is based on experimental LogP (octanole/water) values (P is the repartition coefficient of a molecule between 1-octanol and water) and is is related to ΔG° because a measure of an equilibrium (ΔG°=−2.303RTLogP). It scores the energy interactions between two or more molecules taking into account both contributions to the total free energy, ΔH° and ΔS°.

In the case of mycotoxins and, more in general, food components, we applied this new paradigm to the a set of 12 molecules, six crystallographic complexes and six complexes for which we have only the experimental binding value without crystal structures (see Table 1). The molecular modelling protocol used was born to predict the energetic in biomolecular associations based on logP. The forces involved in a biomecular association are the same involved in an organic host-guest interaction, hydrogen bonds, dipole-dipole interactions, vdW interactions, hydrophobic-hydrophobic interactions.

Sometimes water is the most important intermediate subject involved as solvent or as hydrogen network mediator and, for the specific case of mycotoxins included within the cyclodextrin cavity, it is implied in the quenching fluorescence signal.

CDs are well-known enhancers of toxin fluorescence. For this reason, we considered some major mycotoxins like aflatoxin B1, ochratoxin, zearalenone, α and βzearalenol. Moreover, we also decided to consider resveratrol(63,64) a well-known bioactive compound responsible for important antioxidant effects in food and often used as nutraceutical. All these compounds show a natural fluorescence and have been previously studied for their ability to form complexes with β–CD.

**Figure 1:**
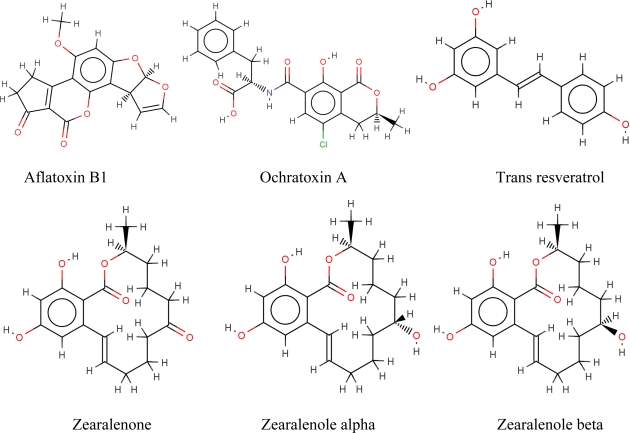
Structure of the six ligands considered in the present study.

Depending on the inclusion direction, βCD can enhance or quench the fluorescence signal. In fact the mechanism of binding/quenching exerted by βCD can act in two different ways. As an example, consider two opposite case, aflatoxin B1 and ochratoxin [70]. In the former case the fluorescent group is deeply included within the CD cavity which protects the group from water quenching, while in the latter case, ochratoxin exposes the fluorescent group to the water and βCD can include it, but not protect against water quenching (see Figure 1). Therefore, the receptor must at times be functionalized to protect the fluorescent side. To make the synthesis more profitable, however, careful simulation analysis must be carried out to define the most useful cyclodextrin modifications.

### 7.1. Methodology

The computational procedure has been tested by applying it to six non redundant crystal structures of βCD ligand complexes retrieved from Cambridge Crystallographic Database (CSD). β-CD was chosen for geometrical reasons (they show the same chemical and geometrical complementarity with β-CD), they are the best host toward the class of ligand we consider. The following complexes were downloaded (65,66): complex of β-CD with phenyalanine (code AGAZOX), complex of β-CD with naphthyloxy acetic acid (code ODEJOW), complex of β-CD with benzyl alcohol (code DEBGOG), complex of β-CD with diclofenac (code HEHJEJ), complex of β-CD with 4 hydroxy-azo-benzene (code FODBEG), complex of β-CD with benzoic acid (code TAFZEG). All these complexes were saved in the Tripos mol2 format using Mercury. SYBYL was used to check the coordinates, to delete co-crystallized water molecules, to separate the β-CD from the ligand and add missing hydrogen atoms when necessary, to rename the atom types and to avoid steric clashes. Added hydrogen atoms were energy-minimized using the Powell algorithm, with a convergence gradient of 0.5 Kal·mol^−1^ for 1500 cycles. This procedure affected only non-experimentally detected hydrogen atoms.

For each of these complexes, HINT scores were evaluated and ΔG° predicted, then the ligand was extracted and re-docked into the βCD cavity using GOLD docking package, obtaining 50 docking poses. The docking was performed assigning full ligand flexibility considering the host, β-CD, as rigid.

All the 50 docking poses generated by GOLD for each guest molecule and ranked with the internal scoring function GoldScore, were re-scored with HINT and the best Hintscore position shows a good relationship between ΔG° and Hintscore as illustrated in Figure 1.

Due to the lack of a wide set of β-CD-ligand complexes in Cambridge Database, it was not possible to test the accuracy of this computational approach for this class of compounds as done for protein-ligand or protein-DNA complexes [72,73]. As non covalent interactions between host and guest are the same as those involved in biomolecular associations, we assumed 500 Hintscores as equivalent to 0.5 Kcal/mol, as demonstrated for protein-ligand complexes dataset regression [71].

Then LogP, Hintscore and ΔG° were calculated for these complexes. The LogP of molecule was calculated using semi-essential, unsaturated C only and via bond options and this value was used to calculate the HINT score.

### 7.2. Results

**Figure 2:**
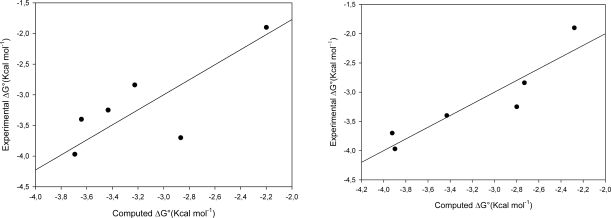
(a) Computed vs Experimental ΔG° values of CSD complexes R = 0,7699, Rsqr=0,5927 (b) Computed vs Experimental ΔG° values of CSD complexes after docking R=0,9200, Rsqr=0,8465.

The first graph in Figure 2(a) shows the good agreement between the experimental ΔG° and estimated ΔG° (using Hintscore) for six crystallographic complexes from Cambridge Structural Database, while the second shows the good agreement when guest compounds were extracted from β—Cyclodextrin and then re-docked using GOLD and Goldscore plus HINT for the energy evaluation.

These results indicate that the docking procedure is also suitable for these kinds of complexes, where the leading association forces are the same as in those biomolecular complexes for which the protocol has been extensively validated [64].

The second graph reported in Figure 2(b) shows the very good agreement between experimental ΔG° and estimated ΔG° (using Hintscore) for six β-CD-guests complexes for which crystal structure does not exist at present (see Table 2).

These molecules are of great interest for food safety and quality and the understanding of the mechanism of binding is important to establish the right or functionalized β–CD which can be used for the development of the best chemosensor to detect mycotoxins at very low concentrations.

**Figure 3:**
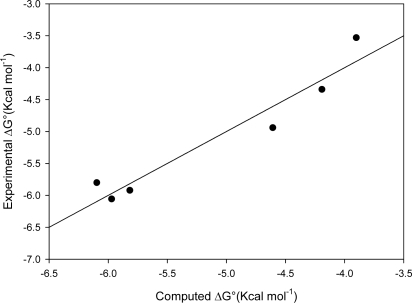
Computed vs Experimental ΔG° values of dataset R =0,9627, Rsqr=0,9269.

The six molecules presented in this paper (see Figure 1), aflatoxin B1, ochratoxin A, resveratrol, zearalenone and α and β-zearalenol are representative of the typical problems faced in modeling inclusion complexes. The docking/scoring procedure applied to a previous test set has been applied to this data set. The graph in Figure 3 shows the good agreement between estimated and experimental ΔG°.

**Figure 4:**
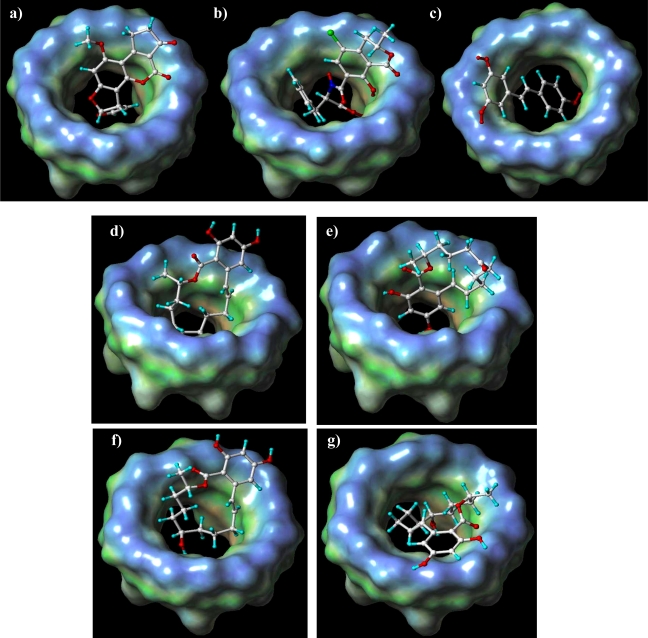
Docking results for a): AF1 and β-CD; b): OTA and β-CD; c): RES and β-CD; d),e): ZEN and β-CD; f): α-ZEN and β-CD; g): β-ZEN and β-CD. Guests are represented with atoms in color code and cyclodextrin solid contour represents polar/hydrophobic surface.

AFB1 is included with the fluorescent part of the molecule, thus β–CD can protect the molecule from the quenching exerted by water. OTA is included in reverse mode, with the fluorescent part unprotected from the water quenching. This is due to a different hydrophobic/polar distribution on the molecule surface. For resveratrol, the inclusion favours the fluorescence phenomenon because it interacts using the phenol side of the molecule. The benzene ring interacts with the hydrophobic cavity and the oxidryl is outside of the cavity, at the bottom, making hydrogen bonds with lower rim functional groups. These models completely explain experimental data from fluorescence analysis (67- 69) which shows an improvement of the signal in case of AFB1 and resveratrol instead of a decreasing for OTA because the fluorescent group is water exposed (See Figures 4a-c).

**Figure 5:**
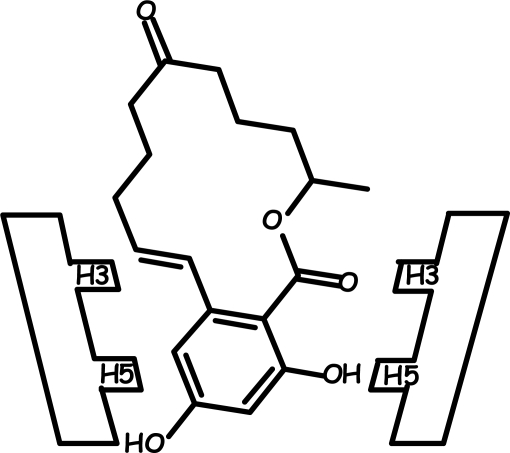
β-CD-zearalenone complex model from NMR data.

Furthermore, polar/hydropobic maps of AFB1 and OTA (Figures 4h,i) show good complemetarity with the β–CD cavity. Zearalenone and zearalenols are a different case study. The models predict two possible solutions for zearalenone with the same energy (Figures 4d, e): one with the benzene ring deep within the cavity and the other one with the aliphatic side deep within the cavity. The docking solutions suggest electrostatic interactions between carbonyl groups at the top and the bottom of β–CD in both solutions. Fluorescence enanchement and NMR analysis (unpublished data, Dr. Roberto Pela, Ph.D. Thesis, “Modified and unmodified β-CDs as mycotoxins receptors”, Università degli Studi di Parma, 2008.) suggest that zearalenone could be included with the benzene ring but the experimental data do not allow us to establish if the stoichiometry of the adduct β–CD/zearalenone is 1:1 or 2:1. Although the NMR data suggest a 1:1 complex, the strong fluorescence enhancement may be in agreement with a stoichiometry β–CD:zearalenone 2:1, where the second binding shows a very fast kinetic not detectable by NMR measurements. This hypothesis is in agreement with the double docking solution. However, the structure suggested by NMR data and reported in Figure 5 is in total agreement with the docking solution reported in Figure 4e.

For α and β zearalenole the modeling solutions are always with the benzene ring outside the cavity. For these molecules experimental data give a different fluorescence enanchement that suggests the different position of the oxidryl group lead the formation of hydrogen bonds with the upper rim in lack of NMR experiments (Figures 4f, g).

In this case the well known limits of docking packages make blind our scoring functions HINT that is not able to discover solutions neglected by GOLD. A possible solution is to modify the force field of the docking software that is normally conceived for biomolecular associations, especially for protein-ligand complexes.

Nevertheless, modeling may be fundamental in developing new modified cyclodextrins to better include some compounds, i.e. OTA, adding functional groups to the upper rim to protect from the quenching by water molecules.

## 8. Conclusions and future perspectives

Due to the increasing number and the variety of chemical structures, biosynthetic origins, toxicological effects and fungal sources, mycotoxins are considered the most important chronic dietary risk factor, above synthetic contaminants, plant toxins, food additives or pesticide residues. Thus, efforts should be made to react quickly and appropriately when faced with new outbreaks of mycotoxicosis.

While traditional mycotoxin analysis has been performed using mainly chromatographic techniques, which provide high sensitivity and accuracy, emerging techniques especially focus on developing fast screening assays, able to perform multi-toxin measurements. Although some MIP-based strategies have been developed, immunoassay-based strategies are the most common.

Nonetheless, the development of chemosensing methods based on biomimetic are advisable due to their stability, low costs and lack of ethical implication. The new approach proposed, based to the integration of molecular modelling and spectroscopic measurements, has been successfully applied at the beginning to understand the different fluorescent behaviour of AFB1 and OTA when complexed with β–CD in lack of structural informations.

In this case it has been applied to a set of organic molecule of most importance for food safety with the aim to drive sinthetic routes to develop new functionalized β–CD able to capture these compound protecting the fluorescent groups necessary for the fluorescence detection. Future development are in order to prepare a new functionalized β–CD specific to protect OTA from water quenching.

## Figures and Tables

**Table 1:** Calculated ΔG° for CSD complexes, ΔG° for CSD complexes after redocking and ΔG° experimental.

CSD Code (molecule complex with beta cyclodextrin)	Calculated ΔG°(Kcal mol^−1^) for CSD complexes	Calculated ΔG°(Kcal mol^−1^) for CSD complexes after docking	Experimental ΔG°(Kcal mol^−1^)
AGAZOX (Phenyalanine)	−2,1995	−2,2799	−1.9000
FODBEG (4-hydroxyazobenzene)	−3,6929	−3,8979	−3.9700
TAFZEG (Benzoic acid)	−3,2255	−2,7297	−2.8400
DEBGOG (Benzyl alcohol)	−3,4333	−2,7998	−3.2500
HEHJEJ (Diclofenac)	−3,6415	−3,4302	−3.4000
ODEJOW (Naphthyloxy acetic acid)	−2,8673	−3,9226	−3.7000

**Table 2:** Experimental K_d_, experimental ΔG° and predicted ΔG° for six new ligands.

Molecules	K_d_ cal	ΔG° experimental (Kcal mol^−1^)	ΔG° Computed (Kcal mol^−1^)
Aflatoxin	2.5−03	−3.530	−3.902
Ochratoxin	6.3 -04	−4.344	−4.192
Resveratrol	2.3−04	−4.910	−4.608
zearalenone	5.3−05	−5.809	−6.097
zearalenole-alfa	3.5−05	−6.056	−5.971
zearalenole-beta	4.4−05	−5.912	−5.816
